# Social factors in childhood and risk of depressive symptoms among adolescents – a longitudinal study in Stockholm, Sweden

**DOI:** 10.1186/s12939-014-0096-0

**Published:** 2014-11-11

**Authors:** Therese Wirback, Jette Möller, Jan-Olov Larsson, Maria Rosaria Galanti, Karin Engström

**Affiliations:** Department of Public Health Sciences, Karolinska Institutet, Tomtebodavägen 18A, 171 77 Stockholm, Sweden; Department of Women’s and Children’s Health, FoUU BUP, q3:4 Astrid Lindgren Children’s Hospital, 171 77 Stockholm, Sweden; Centre for Epidemiology and Community Medicine, Stockholm County Council, 171 77 Stockholm, Sweden

**Keywords:** Socioeconomic status, Depressive symptoms, Adolescence, Social inequalities, Longitudinal

## Abstract

**Background:**

In Sweden, self-reported depressive symptoms have increased among young people of both genders, but little is known about social differences in the risk of depressive symptoms among adolescents in welfare states, where such differences can be less pronounced. Therefore, the aim was to investigate whether multiple measures of low social status in childhood affect depressive symptoms in adolescence. A secondary aim was to explore potential gender effect modification.

**Methods:**

Participants were recruited in 1998 for a longitudinal study named BROMS. The study population at baseline consisted of 3020 children, 11–12 years-old, from 118 schools in Stockholm County, followed up through adolescence. This study is based on 1880 adolescents answering the follow-up survey in 2004, at age 17–18 (62% of the initial cohort). Parental education, occupation, country of birth, employment status and living arrangements were reported at baseline, by parents and adolescents. Depressive symptoms were self-reported by the adolescents in 2004, using a 12-item inventory. The associations between childhood social status and depressive symptoms in adolescence are presented as Odds Ratios (OR), estimated through logistic regression. Gender interaction with social factors was estimated through Synergy Index (SI).

**Results:**

Increased risk of depressive symptoms was found among adolescents whose parents had low education (OR 1.8, CI = 1.1-3.1), were unskilled workers (OR 2.1, CI = 1.2-3.7), intermediate non-manual workers (OR 1.8, CI = 1.0-3.0), or self-employed (OR 2.2, CI = 1.2-3.7), compared to parents with high education and high non-manual work. In addition, adolescents living exclusively with one adult had an increased risk compared to those living with two (OR 2.8, CI = 1.1-7.5), while having foreign-born parents was not associated with depressive symptoms. An interaction effect was seen between gender and social factors, with an increased risk for girls of low-educated parents (SI = 3.4, CI = 1.3-8.9) or living exclusively with one adult (SI = 4.9, CI = 1.4-6.8).

**Conclusions:**

The low social position in childhood may increase the risk of depressive symptoms among adolescents even in countries with small social differences and a highly developed welfare system, such as Sweden. Girls with low educated parents or living exclusively with one adult may be particularly vulnerable. This knowledge is of importance when planning preventive interventions or treatment.

**Electronic supplementary material:**

The online version of this article (doi:10.1186/s12939-014-0096-0) contains supplementary material, which is available to authorized users.

## Background

Mental ill-health is the largest public health problem in high-income countries [1], the second largest contributor to years lived with disabilities (DLL) in the world and the number one problem among 10–14 year olds in Canada and the US [2]. Girls are most often found to be at a higher risk of depressive symptoms than boys [3-9] though the opposite has also been found [10]. In Sweden, self-reported depressive symptoms have increased among young people of both genders in the past two decades [1,11]. Also, the prevalence of adolescents treated for depression has increased [12]. This is especially worrisome given that depression in adolescence, including subclinical depression [13], is a risk factor for a clinical depression later in life [14] as well as for other types of mental ill-health [15], substance abuse [16], suicide [17] and low educational attainment and unemployment [9,15,17].

Most health problems are more common among groups with lower socioeconomic status (SES) [18]. Social and economic factors can thus, along with psychosocial and genetic factors, be expected to play a role in developing symptoms of mental ill-health in adolescents [9]. Exposure to disadvantageous social and material family conditions, such as low educational level, unemployment, lack of resources and lack of time, can be hypothesized both to increase mental distress among children and to decrease possibilities for parents to afford, demand and receive good health care for their children.

A frequently used way of representing differences in health is by an individual’s, or in the case of children, their parents’ socioeconomic status (SES), including income, occupation and education. The relationship between SES and depressive symptoms is not straightforward, as illustrated by studies both among adolescents [3,4,6,8,10,19-28] and adults [21,26,29-34]. Comprehension of the field is hampered by heterogeneity of the indicators used to define SES (e.g. income, education and occupational class), illness severity and definitions of depressive symptoms (including clinical depression as well as self-reported) as well as by differential adjustment for confounding factors. With regard to studies on adolescents, most found at least one indicator of low SES (based on parent or family information), linked to the risk of depressive symptoms, [3,4,6,8,10,19-25], but in some cases an opposite association was found for another indicator of SES [3,8,10]. However, studies employing several measures of SES are rare, and the employment of unrefined measures, e.g. dichotomized exposures, is common [3,4,10]. In addition, many of the studies showing associations between depressive symptoms in adolescence and parental income or education used a cross-sectional design, generating problems of reversed causality [10,22,23,25].

Other social factors in childhood shown to be correlated with depressive symptoms in adolescence or later in life are e.g. parental geographical origin [11], financial difficulties [16,35], parental unemployment [16] and living with a single parent [10,33].

Gender differences in the association between parental SES and depressive symptoms in adolescence have rarely been studied, though Reiss et al. [28] concludes in a recent systematic review that there is inconsistency in gender patterns in SES and mental health problems.

Little is known concerning social differences in the risk of depressive symptoms among adolescents in the form of welfare states typical of the Nordic countries, where social differences are likely to be less pronounced. In fact, most studies were conducted in the US [4,20,23,25,34], and some in different European countries, e.g. Spain [10], Hungary [8], Holland [19,24] and the UK [24]. Only two recently published studies from the Nordic countries were identified, both from Finland [3,6]. Elovainio et al. [3] showed that manual parental occupation but not low parental income, was associated with self-rated depressive symptoms among adolescents. Paananen et al. [6] found that low parental education and manual occupation increased the risk of mental disorders.

Studies from Sweden concerning social status and depressive symptoms in adolescence would importantly contribute to expanding the limited knowledge on social inequalities in mental health in this age group. This is especially important due to the increase in depressive symptoms reported by this group. Therefore, the aim was to investigate whether multiple measures of low social status of the family are longitudinally associated with depressive symptoms in adolescence. A secondary aim was to explore potential gender effect modification. This was made possible by using information from a unique, longitudinal, population-based sample of adolescents.

## Methods

### Material

This study is based on BROMS (acronym in Swedish for Children’s Smoking and Environment in Stockholm County), a cohort study designed to investigate tobacco use among adolescents in Stockholm, Sweden. The BROMS study started in 1998 when 6 294 adolescents in 5^th^ grade from 118 schools in Stockholm were invited to participate. Among those, 3 020 adolescents from 91 schools participated (48%). Mean age of the participants in 1998 was 11.6 years [36]. In total, eight surveys have been conducted with the same adolescents, once every year, starting in 5^th^ grade, with a pause the first year after compulsory school (1998–2005) and one five years later (2010) [36,37]. In addition, a survey was also conducted among the children’s parents or other guardian at baseline in 1998. The BROMS cohort is a rich source of information about health and living circumstances among adolescents, and comprises information regarding a variety of social and health-related factors, including depressive symptoms.

### Study population and design

In this study, the cohort was restricted to adolescents answering both the baseline survey in January 1998, at the age of 11–12 years, and the follow-up survey in January 2004, at the age of 17–18 years, whose parents or other guardian had answered the parental questionnaire in 1998. This corresponds to 2 622 adolescents, i.e. 87% of the eligible cohort members. Finally, due to missing values in exposure and/or outcome variables, the final analyses are based on 1880 adolescents (62% of the initial cohort).

The Huddinge Hospital Ethical Committee (10–97) and the Stockholm Regional Ethical Review Board (2013/1896-32) approved the study. Informed consent was obtained from the guardians of all participants in the study.

### Social factors

In this study, two indicators of socioeconomic status (SES) were used – parental educational level and occupational class. Occupation refers more to prestige attached to social position and education is more related to health literacy. In addition, we included parental country of origin and two factors likely to affect the economy of the family – parental employment and living exclusively with one adult. Besides measuring economic standard, parental employment and living exclusively with one adult can be considered as global indicators of the psychosocial circumstances of the family [38].

Most information on social factors was obtained from the baseline parental survey in 1998, with the exception of who the child lives with, which was based on children’s report in the same year. The parental survey was filled in by either the child’s mother, father or other guardian (hereafter referred to as parent), including information regarding him- or herself and the other parent/guardian. *Parental educational level* was based on number of school years completed by the parent with highest education. It was categorised into three groups; low (compulsory school, 0–9 years), intermediate (upper secondary education, 10–12 years) and high (post-secondary education, 13 years or above). *Parental occupational class* was based on the highest occupation reported for both parents, or on the only parent’s occupation, according to dominance principles [39]. It was analysed in seven categories according to Statistics Sweden’s socioeconomic classification [40]; unskilled worker, skilled worker, lower non-manual, intermediate non-manual, higher non-manual, self-employed and other. *Parental country of birth* was categorised into “Sweden” or “other”. Adolescents were considered as having parents born outside Sweden if both parents or the single parent were born outside Sweden. *Parental employment* was based on a question regarding current employment in 1998. Adolescents who had at least one employed parent, part- or full-time, were categorised as having employed parents. *Living arrangements* was defined by adolescents reporting who they lived with and was categorised into living exclusively with one adult or living with two or more adults. Children with separated parents, living with both parents equal time or only occasionally with one, and children living with only one parent who is cohabiting with another adult were categorised as living with two or more adults. An “adult” was in this case defined as mother, father, stepfather/mother, grandfather/mother or foster-father/mother that the adolescent lived with.

### Depressive symptoms

Depressive symptoms were assessed in 2004, with a 12-item inventory (see Additional file 1). The adolescents were asked to rate the frequency of occurrence of certain mood or behaviours in the past 30 days. Response options were: never, sometimes, often or very often. The inventory has not previously been validated, but corresponds to a large extent criteria set in Diagnostic and Statistical Manual of Mental Disorders, version IV (DSM-IV) of depression [41]. Depressive symptoms were then categorised in two different ways. First a dichotomous DSM-IV criteria-based variable was created, where presence of depression was defined as closely as possible to the original diagnostic criteria. Having chosen the option ”often“ either for the item “felt unhappy and sad” or for the item ”felt tired and have lack of interest”, and on additionally four items was considered indicative of depressive symptoms. The second variable, also dichotomized, was based on a summary score for all 12 items. Answers were coded as 0 (never), 1 (sometimes), 2 (often) or 3 (very often) with a total score ranging from 0 to 30. Dichotomization was set to the decile with most severe depressive symptoms, which equalled a score of 17 or higher. Because of ties in the score, 11.2% of the adolescents were allocated to the group with depressive symptoms. This measure will be referred to as “Score 17”. Cronbach’s α [42] showed a consistency for the 12-item inventory of 0.87.

### Statistical analysis

Odds ratios (OR), with corresponding 95% confidence intervals, calculated through logistic regression models, were used to estimate the association between family’s social status at age 11–12 and self-reported depressive symptoms later in adolescence (17–18 years old). First, crude ORs were calculated for each social factor and each of the two outcome variables. Secondly, adjustments were done for a) gender, and b) mutually for all social factors, to distil the effect of each of them. Thirdly, a Synergy Index (SI) [43,44] was calculated in order to assess additive interactions between gender and social factors. To this end, all independent variables were dichotomized. Parental occupational class was divided into manual and non-manual workers (where self-employed were categorised as manual and “others” were excluded) and parental educational level was divided into low vs. intermediate/high.

To be able to compare results from crude models with adjusted, participants with missing data (for the proportion of missing values, see Table 1) for one or more variables were excluded from all analyses (listwise deletion), resulting in a final analytical sample of 1 880 adolescents. The outcome variable was set to missing when more than one scale item was unanswered or when lacking information on one scale item made the determination of depressive symptoms impossible. Information on a given social factor was treated as missing when information was missing for both parents or for legal guardians. In the case of living arrangements, missing values corresponded to lack of information reported by the adolescent. To examine the impact of the missing data, crude ORs were calculated for each social factor, including all respondents with available information. All analyses were conducted using SAS version 9.2 and 9.3 (SAS Institute Inc. Cary, N.C., USA).

**Table 1:** **Socio-demographic characteristics of the study participants, n = 2622**

	**Total**	**Depressive symptoms**	
	***n = 2622***	**Score 17** ***n = 292***	**DSM-IV criteria- based ** ***n = 167***
	**Column %**	**Row %**	**Row %**
**Gender**			
Girl	50.1	16.8	9.4
Boy	49.9	5.6	3.4
*Missing*	*0*	*0*	*0*
**Parental education**			
High	57.6	10.7	6.3
Intermediate	30.0	10.7	6.8
Low	4.5	15.9	7.7
*Missing*	*7.9*	*12.4*	*5.1*
**Parental SES**			
High non-manual workers	12.8	7.3	5.6
Intermediate non-manual workers	20.8	10.4	7.1
Lower non-manual workers	14.7	10.4	5.5
Skilled workers	11.6	10.3	6.3
Unskilled workers	18.0	13.8	7.7
Self-employed	6.7	13.1	7.8
Other	2.8	11.1	7.0
*Missing*	*12.7*	*13.5*	*5.1*
**Parental country of birth**			
Sweden	73.4	10.1	6.2
Outside Sweden	11.3	12.8	5.1
*Missing*	*15.3*	*15.1*	*8.5*
**Living arrangements**			
Lives with two or more adults	98.2	11.0	6.3
Lives exclusively with one adult	1.8	21.7	8.7
*Missing*	*0.6*	*13.3*	*14.3*
**Parental employment status**			
Employed	86.2	10.7	6.6
Unemployed	13.8	14.4	5.6
*Missing*	*0*	*0*	*0*

## Results

The characteristics of the study participants are presented in Table 1. More than half of the children had parents with high education and more than a third had parents in high or intermediate non-manual occupations. The majority had Swedish-born parents and almost all lived with at least two adults, including those in joint physical custody.

### Social factors and depressive symptoms, Score 17

**Figure 1:**
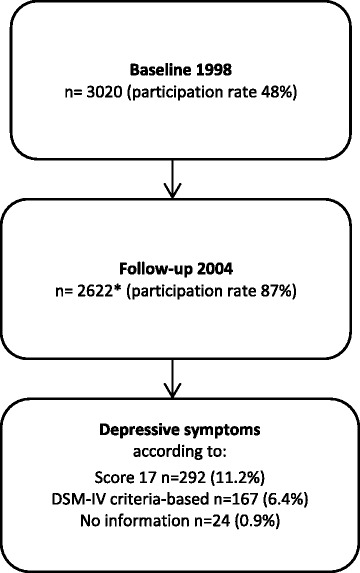
**Study population including proportion with depressive symptoms, using two different measures.** *Of those, 1880 remained in the final analytical sample; 742 were not included due to partially missing answers.

The prevalence of depressive symptoms measured as Score 17 was 11.2% (Figure 1). Crude and adjusted ORs for depressive symptoms measured as Score 17 are shown in Table 2. In unadjusted analyses, adolescents with parents with the lowest educational level were at increased risk of depressive symptoms (OR = 1.8, CI = 1.1-3.1) compared to those with highly educated parents. There were no differences in risk between intermediate and high parental educational level. Adolescents with parents in the category of unskilled workers had twice as high risk of depressive symptoms (OR = 2.1, CI = 1.2-3.7) as those from high non-manual workers. A similar increased risk was found for those having self-employed parents (OR = 2.2, CI = 1.2-3.7). Increased risk (OR = 1.8, CI = 1.0-3.0) was also seen for intermediate non-manual workers.

**Table 2:** **Odds ratios of depressive symptoms in adolescence, measured by Score 17, by social factors, n = 1880**

**Social factors**	**Crude OR (95% ** **CI)**	**Adjusted for gender OR (95% ** **CI)**	Mutually adjusted* OR (95% **CI)**
**Gender**			
Boy	1.0		
Girl	3.4 (2.6-4.5)		
**Parental education**			
High	1.0	1.0	1.0
Intermediate	1.0 (0.7-1.4)	1.0 (0.7-1.4)	0.9 (0.7-1.3)
Low	1.8 (1.1-3.1)	1.7 (1.0-2.8)	1.5 (0.9-2.5)
**Parental occupation**			
Higher non-manual workers	1.0	1.0	1.0
Intermediate non-manual workers	1.8 (1.0-3.0)	1.8 (1.0-3.2)	1.8 (1.0-3.2)
Lower non-manual workers	1.7 (0.9-3.1)	1.7 (0.9-3.0)	1.6 (0.9-2.9)
Skilled workers	1.4 (0.7-2.7)	1.4 (0.7-2.7)	1.3 (0.7-2.6)
Unskilled workers	2.1 (1.2-3.7)	2.1 (1.2-3.7)	2.0 (1.1-3.6)
Self-employed	2.2 (1.2-3.7)	2.1 (1.0-4.2)	1.9 (0.9-3.9)
Others	1.8 (0.7-4.6)	1.7 (0.7-4.2)	1.3 (0.5-3.5)
**Parental country of birth**			
Sweden	1.0	1.0	1.0
Outside Sweden	1.3 (0.8-2.0)	1.2 (0.8-1.9)	1.1 (0.7-1.7)
**Living arrangements**			
Lives with two or more adults	1.0	1.0	1.0
Lives exclusively with one adult	2.8 (1.1-7.1)	3.0 (1.1-7.8)	2.8 (1.1-7.5)
**Parental employment status**			
Employed	1.0	1.0	1.0
Unemployed	2.6 (1.1-6.1)	2.1 (0.9-5.0)	1.8 (0.7-4.5)

*All social factors are adjusted for each other and for gender.

Approximately 11% of the adolescents had both parents born outside Sweden (Table 1). Having foreign-born parents was not associated with risk of depressive symptoms, measured as Score 17. Living with exclusively one adult was rare (2%) but doing so was associated with an almost three-fold higher risk of depressive symptoms (OR = 2.8, OR = 1.1-7.1). Having unemployed parents was also associated with a higher risk of depressive symptoms (OR = 2.6, CI = 1.1-6.1).

After adjustment for gender, all associations but one (the increased risk of having unemployed parents) were confirmed. In the mutual adjustment for all social factors, a significant association with depressive symptoms was still evident for living with exclusively one adult (OR = 2.8, CI = 1.1-7.5), and for having parents being unskilled workers (OR = 2.0, CI = 1.1-3.6) or intermediate non-manual workers (OR = 1.8, CI = 1.0-3.2). For all other associations detected in the gender-adjusted analysis, elevated point estimates remained, albeit slightly decreased and no longer statistically significant.

A sensitivity analysis, where crude ORs were calculated including all respondents with available information (1975–2622 respondents; results not shown), showed slightly lower point estimates, but did not differ significantly from ORs based on the sample of 1 880 used in Table 2.

### Social factors and depressive symptoms, DSM-IV criteria-based

The prevalence of DSM-IV criteria-based depressive symptoms was 6.4% (Figure 1). Overall, results from the DSM-IV criteria-based outcome variable (Table 3) did not contradict the results for Score 17 (Table 2). However, using the DSM-IV criteria-based outcome yielded weaker and more imprecise associations.

**Table 3:** **Odds ratios of depressive symptoms in adolescence, measured as DSM-IV criteria-based, by social factors, n = 1880**

**Social factors**	**Crude OR (95% ** **CI)**	**Adjusted for gender OR (95%** **CI)**	Mutually adjusted* OR (95% **CI)**
**Gender**			
Boy	1.0		
Girl	3.0 (2.1-4.2)		
**Parental education**			
High	1.0	1.0	1.0
Intermediate	1.1 (0.8-1.7)	1.1 (0.8-1.7)	1.1 (0.8-1.7)
Low	1.2 (0.6-2.5)	1.2 (0.6-2.5)	1.2 (0.6-2.5)
**Parental occupation**			
Higher non-manual workers	1.0	1.0	1.0
Intermediate non-manual workers	1.6 (0.8-3.0)	1.6 (0.8-3.0)	1.6 (0.8-3.0)
Lower non-manual workers	1.2 (0.6-2.4)	1.2 (0.6-2.4)	1.1 (0.5-2.3)
Skilled workers	1.0 (0.5-2.1)	1.0 (0.5-2.1)	0.9 (0.4-2.1)
Unskilled workers	1.4 (0.7-2.8)	1.4 (0.7-2.8)	1.3 (0.7-2.7)
Self-employed	1.1 (0.5-2.7)	1.1 (0.5-2.7)	1.0 (0.4-2.6)
Others	1.5 (0.5-4.4)	1.5 (0.5-4.4)	1.4 (0.4-4.3)
**Parental country of birth**			
Sweden	1.0	1.0	1.0
Outside Sweden	1.0 (0.5-1.8)	1.0 (0.5-1.8)	1.0 (0.5-1.8)
**Living arrangements**			
Lives with two or more adults	1.0	1.0	1.0
Lives exclusively with one adult	2.1 (0.6-7.3)	2.1 (0.6-7.3)	2.1 (0.6-7.2)
**Parental employment status**			
Employed	1.0	1.0	1.0
Unemployed	1.3 (0.4-4.4)	1.3 (0.4-4.4)	1.3 (0.4-4.4)

*All social factors are adjusted for each other and for gender.

As was the case for depression symptoms measured by Score 17, crude ORs calculated including all respondents (n = 1975-2622) with available information were similar (results not shown) to those based on listwise deletion in Table 3.

### Gender effect modification of social factors

Being a girl entailed substantially higher risk for depressive symptoms compared to being a boy, both when measured as Score 17 (OR = 3.4, CI = 2.6-4.5; Table 2) and when using the DSM-IV criteria-based variable (OR = 3.0, CI = 2.1-4.2; Table 3). Table 4 shows synergy effects, from interaction analyses, between gender and other social factors on depressive symptoms according to Score 17. A significant synergy effect was found for being a girl and having parents holding a low educational level (SI = 3.4, CI = 1.3-8.9) or living exclusively with one adult (SI = 4.9, CI = 1.4-6.8). Parental occupational level, parental country of birth and parental employment status showed only additive effects.

**Table 4:** **Odds ratios and Synergy Index between gender and social factors on depressive symptoms, score17, n = 1880**

**Social factors**	**Male**		**Female**		**Synergy index**
	**Unexposed to social factor**	**Exposed to social factor**	**Unexposed to social factor**	**Exposed to social factor**	
	**OR (95% ** **CI)**	**OR (95% ** **CI)**	**OR (95% ** **CI)**	**OR (95% ** **CI)**	**SI (95% CI)**
**Parental education Low vs. high**	1.0	0.6 (0.2-2.7)	2.8 (2.1-3.7)	5.8 (3.3-10.2)	3.4 (1.3-8.9)
**Parental occupation Manual vs. non-manual**	1.0	1.1 (0.6-1.9)	2.4 (1.8-3.1)	2.8 (1.8-4.3)	1.2 (0.6-2.7)
**Parental country of birth Foreign vs. Sweden**	1.0	1.0 (0.5-1.9)	2.2 (1.7-2.9)	2.6 (1.6-4.1)	1.3 (0.5-3.2)
**Living arrangements One vs. two or more**	1.0	0.8 (0.1-5.6)	3.2 (2.4-4.3)	10.6 (4.4-5.3)	4.9 (1.4-6.8)
**Parental employment status Unemployed vs. employed**	1.0	1.2 (0.6-2.3)	3.3 (2.4-4.5)	5.0 (3.2-7.7)	1.6 (0.9-2.9)

## Discussion

To our knowledge, this is the first Swedish study conducted with adolescents, using a longitudinal, population-based design with several predictors of SES in relation to depressive symptoms. Low parental occupational class and low parental education as well as parental unemployment were found to impact on the risk of depressive symptoms among adolescents. Living exclusively with one adult almost tripled the risk of depressive symptoms. Moreover, it was found that girls were particularly vulnerable to depressive symptoms when living in families with low educated parents or living exclusively with one adult. In the present study, point estimates were overall shown to be lower when calculated with a DSM-IV criteria-based measure rather than with a summarized score.

Differences in health may partly occur because exposure to exogenous risk factors varies between different groups in society. Lower socioeconomic groups are more likely to be exposed to economic hardship, the burden of large families and serious conflicts during children’s upbringing [45]. The purpose of a welfare state is to minimize both the exposure to such environments and their potential consequences in terms of susceptibility to health problems [45]. The Swedish welfare system is built on efforts to achieve full employment, relatively generous benefit levels, high-quality public care services, and relatively small inequalities between genders as well as between different socioeconomic groups. Nevertheless, our results show that Swedish adolescents from socioeconomically disadvantaged families, measured in different ways, were at higher risk of depressive symptoms than those from more advantaged backgrounds. These results are in line with two studies conducted in Finland, a country with a welfare system similar to Sweden’s [3,6]. Looking at relative differences between socioeconomic groups could partly explain the magnitude of differences found, knowing that low overall prevalence might increase relative differences. The overall prevalence of depressive symptoms has been found to be higher in less affluent countries as well as in countries with greater income inequality and weaker redistributive policies [46,47]. Indeed, in an earlier study by Mackenbach et al. [48], it has been shown that relative inequalities in some health outcomes are larger than average in Sweden, even though absolute differences are small [49].

The increased risk for depressive symptoms among adolescents with low parental education was previously reported in several other studies, both from Europe and the US [5,10,21-23,25], including a Finnish study [6]. Exceptions were two European studies; Piko et al. [8], who studied well-being and Huisman [24], who analysed mild depressive symptoms. Most previous studies [3,4,10,21], only comparing manual workers with non-manual workers, showed that adolescents with parents in manual occupations have a higher risk of depressive symptoms. The current study not only confirmed this association, but showed that the increased risk also applies to adolescents whose parents had low and intermediate non-manual work. Interestingly, adolescents with self-employed parents also had an increased risk of depressive symptoms. This was in contrast with an earlier study from Hungary, where the father’s self-employment seemed to decrease the risk of depressive symptoms reported by parents [8]. This discrepancy may be due to an artefact as a result of reporting source of depressive symptoms, but it could also be explained by differences between Hungary and Sweden with regard to different socioeconomic circumstances for the self-employed. A potential explanation for an increased risk among Swedish adolescents with self-employed parents could be the high workload among the self-employed, with consequent lower participation in children’s lives. Paternal absence has been found to explain higher risks of depressive symptoms [50].

The majority of European studies, including one from Finland, found no association between low parental income and depressive symptoms among adolescents [3,8,10], while most American studies [5,20,23,25] did. These differences may partly be due to different financial support systems and welfare state programmes in the specific countries. The Swedish welfare system is relatively strongly redistributive and no major differences could be expected in depressive symptoms due to economic circumstances. Since, in this study, information on parental income was not available, it relied on two factors considered to affect the economy of the family, i.e. parental employment and living arrangements. Results showed an increased risk of depressive symptoms associated with both unemployed parents and living with exclusively one adult. This result is in line with findings from earlier studies [10,32]. However, research has also shown that both unemployment and households with only one adult are associated with other possible aspects known to influence the risk of depressive symptoms, apart from the economic situation [38]. It has been suggested that two-parent families, apart from leading to higher standard of living, also provide more effective parenting, deeper emotional closeness and less stressful life events [51]. Children growing up in two-parent families suffer less frequently from cognitive, emotional and social problems [51]. Conversely, children living with a single parent appear to be at increased risk of psychiatric disease and suicide [38]. Living with only one parent could also indicate witnessing family changes, e.g. separation or divorce, a stressful life event that, according to Storksen et al. [50], is a risk factor for depression. The exposure used in this study is more distilled and includes only those who exclusively live with one adult to better capture the group most economically disadvantaged. Further differentiating between different forms of living arrangements could add important information [52], however such data was unavailable.

In this study, no differences in the risk of depressive symptoms were found between adolescents with Swedish-born and foreign-born parents, which is in line with a Swedish report from the National Institute of Public Health [11]. Many studies have shown a higher risk of depressive symptoms among adult immigrants [32,53,54]. However, it is not clear to what extent this is explained by their own immigration history or by experiences after immigration. In the current study, the large majority of adolescents with foreign-born parents were themselves born in Sweden, therefore an analysis of immigration history at the adolescent level was not possible.

The association of depressive symptoms with most socioeconomic factors persisted even after mutual adjustment for each other, indicating independent associations. The persistence of a significant association with occupational class but not with parental education emphasizes the importance of the indicator used to measure SES [30]. Geyer et al. [55] imply that the indicators are interconnected but should not be used interchangeably, since the cause and effect chain depends on what is being studied. This study did not attempt to explore how different social factors are interconnected with regard to depressive symptoms, however the results reported underline the need of such studies in this field.

As expected, girls in this population sample were at higher risk of depressive symptoms than boys. Contributing to the understanding of the gender-specific risk of depressive symptoms, this study showed that the increased risk associated with low-educated parents and with living exclusively with one adult was more pronounced among girls than among boys. Other studies on gender-specificity have shown inconsistent results [24,25,28]. Some show girls to be more vulnerable [56,57]. For instance, Mendelson [57] found that a subgroup of girls with the lowest parental education and household income had a higher risk of internalized problems than boys. Conversely, some studies report the opposite [24,58,59], for example Huisman et al. [24] which demonstrated that boys in single-parent households had a higher risk of internalized problems than girls. Yet other studies didn’t find any gender-specific risk of depressive symptoms in relation to SES [19,25,60]. This inconsistency might to some extent be explained by different indicators of social factors and mental health. In addition, misclassification of the outcome should be considered as a possible explanation for the differences in findings, since expressions of depressive symptoms have been reported to differ between boys and girls [18].

Both persons with diagnosed depression and those with self-reported depressive symptoms might experience different combinations of depressive symptoms. Sometimes among children, irritability is used as criterion for depression, instead of depressed mood [61]. Also, it has been argued that cut points for depression lack evidence [62]. This indicates that assessing depressive symptoms is complex. In the present study, point estimates were overall lower measured with the DSM-IV criteria-based measure than with the summarized score. A potential explanation is that the summarized score, with a cutoff set to include the 10% with worst mental health, is a more sensitive measure of severe depressive symptoms than DSM-IV criteria-based measure (approx. 6%). Furthermore, anxiety and depression have often shown to be difficult to separate. Olino et al. [63], explain this comorbidity by the combination of similar and diagnosis-specific causes. Since differentiating between disorders is a complex endeavour, and literature on the role of social factors in their occurrence is sparse, a suggestion for future research is to investigate patterns of comorbidity as such.

### Strengths and limitations

The BROMS-cohort provides high-quality longitudinal data during the whole course of adolescence. Depressive symptoms were self-reported, therefore less affected by care-seeking behaviour. This is important since the propensity to seek care may itself be socially determined [64]. In addition, several different indicators of SES as well as other social factors were available, which made it possible to study the effect of social factors on depressive symptoms in greater detail than previously done. Another important strength of the study is the prospective design, which reduced the likelihood of reversed causality, since parental SES and other social factors were assessed before the outcome.

Some selection occurred at the cohort’s recruitment and follow-up, and attrition in both steps could be associated with social factors. While the proportion of children in the cohort under study with both parents living together corresponded well to the national average (71%), [65], parents with college education were overrepresented [36] and foreign-born parents were underrepresented [66], in comparison with the regional average. The most socially vulnerable groups, e.g. undocumented children and children in families with alcohol or drug abuse, are unlikely to be represented at all. The selection might lead to an underestimation of prevalences. There is, however, no reason to believe that this selection may have introduced a bias in the results, since it would not be dependent on the presence of future depressive symptoms in the offspring. Furthermore, by applying listwise deletion, 742 respondents were lost due to missing values on any one variable in the analyses. However, attrition from missing values is not expected to be strongly associated with SES and sensitivity analyses for crude ORs, including all respondents’ available information, showed no significant differences from those where listwise deletion was used.

A further limitation was the inability to adjust for parental history of mental disease. A possible confounding effect of this, however, is not likely to explain the whole excess risk linked to low SES. In fact, the contribution of heredity to major depression is estimated at about 37% [67]. Kestilä et al. [68] found that parental mental health problems increase the risk of psychological distress, however Modin et al. [69] did not. Further, genes and environment may interact [70] and environmental factors may play a significant role even in the onset of disorders that are mainly inherited [71].

Potential misclassification of outcome may have occurred because of inadequate ability among adolescents to recognize and report symptoms, and this misclassification might theoretically differ between socioeconomic groups but particular between boys and girls [18]. However, it has been shown previously that differential reporting according to parental SES is a minor concern [72]. Moreover, symptoms were reported in late adolescence when all subjects had completed compulsory school, thus making major differential misclassification due to literacy problems even more unlikely. In addition, the inventory for the assessment of depression used here has not been previously validated. The inventory broadly corresponds to DSM-IV criteria of depression [41] though all information was self-reported, not diagnostic. In relation to DSM-IV, a couple of questions were added or taken out, e.g. a question on feelings of being “complaining” was added while one question concerning thoughts on death or suicide and one on guilt and restlessness, were taken out. Cronbach’s α [42] showed good internal consistency [73] among the items within the inventory.

Further, this study had insufficient statistical power to closely investigate subgroup differences. Nevertheless, this study revealed an interaction effect between gender and some of the social factors, which underlines the importance of further studies in this area.

## Conclusions

Social factors, such as low parental education, low occupational class, living in a single-parent home or having unemployed parents, are likely to increase the risk of depressive symptoms among adolescents even in societies with a strong welfare system. Girls living with low-educated parents or with exclusively one adult are especially vulnerable. This knowledge should guide preventive interventions to reduce mental health inequality. Continued work is needed to reduce inequalities in depressive symptoms.

## Additional file

Additional file 1:
**Inventory – questionnaire on depressive symptoms.** Translated from Swedish to English.
