# Supplementary material for: Social factors in childhood and risk of depressive symptoms among adolescents – a longitudinal study in Stockholm, Sweden
Source: Int J Equity Health. 2014 Nov 11;13:96. doi: 10.1186/s12939-014-0096-0 (PMC4243322; doi:10.1186/s12939-014-0096-0)
Supplement: Additional file 1: — Inventory – questionnaire on depressive symptoms. Translated from Swedish to English. [file 12939_2014_96_MOESM1_ESM.docx]

Additional file 1. Inventory – questionnaire on depressive symptoms

1. **Has felt grumpy/moody** t**he last month (30 days)**

Never

Sometimes

Often

Very often

1. **Has had difficulties falling asleep or sleeping the last month (30 days)**

Never

Sometimes

Often

Very often

1. **Has felt unhappy or sad the last month (30 days)**

Never

Sometimes

Often

Very often

1. **Has had no strength to do anything the last month (30 days)**

Never

Sometimes

Often

Very often

1. **Has not liked him-/herself the last month (30 days)**

Never

Sometimes

Often

Very often

1. **Has felt tired and taken no pleasure in doing anything the last month (30 days)**

Never

Sometimes

Often

Very often

1. **Has felt that he/she cannot perform as well as others the last month (30 days)**

Never

Sometimes

Often

Very often

1. **Has felt that nothing has gone well for him/her the last month (30 days)**

Never

Sometimes

Often

Very often

1. **Has been eating unusually much the last month (30 days)**

Never

Sometimes

Often

Very often

1. **Has been sleeping unusually much the last month (30 days)**

Never

Sometimes

Often

Very often

1. **Has had difficulties concentrating the last month (30 days)**

Never

Sometimes

Often

Very often

1. **Has skipped meals and been a poor eater the last month (30 days)**

Never

Sometimes

Often

Very often
